# LncRNA BLAT1 is Upregulated in Basal-like Breast Cancer through Epigenetic Modifications

**DOI:** 10.1038/s41598-018-33629-y

**Published:** 2018-10-22

**Authors:** Yoo Jane Han, Sonja M. Boatman, Jing Zhang, Xinxin C. Du, Albert C. Yeh, Yonglan Zheng, Jeffrey Mueller, Olufunmilayo I. Olopade

**Affiliations:** 1 Center for Clinical Cancer Genetics and Global Health and Section of Hematology and Oncology, Department of Medicine, University of Chicago, Chicago, IL 60637 USA; 2 Fred Hutch Cancer Center, University of Washington, Seattle, WA 98109 USA; 3 Department of Pathology, University of Chicago, Chicago, IL 60637 USA

## Abstract

Long-noncoding RNAs (lncRNAs) have been shown to participate in oncogenesis across a variety of cancers and may represent novel therapeutic targets. However, little is known about the role of lncRNAs in basal-like breast cancer (BLBC), the aggressive form of breast cancer with no molecularly defined therapeutic target. To examine whether altered lncRNA expression contributes to the aggressive phenotype characteristic of BLBC, we performed a comparative analysis of BLBC versus non-BLBC using microarray profiling and RNA sequencing of primary breast cancer. We identified RP11-19E11.1 as a significantly up-regulated lncRNA in BLBC tumors and named it Basal-Like breast cancer Associated Transcript 1 (BLAT1). Analysis of pan-cancer datasets showed the highest expression of BLAT1 in BLBC tumors compared to all other cancers. Depletion of BLAT1 in breast cancer cells led to significantly increased apoptosis, partly because of accumulation of DNA damage. Mechanistically, BLAT1 expression is regulated at the epigenetic level via DNA methylation at CpG islands in the promoter. Concordantly, patients harboring tumors with BLAT1 hypomethylation showed decreased overall survival. Our results suggest that increased expression of BLAT1 via CpG site hypomethylation may contribute to the aggressive phenotype of BLBC, raising a possibility of new biomarkers for prognosis of aggressive BLBC tumors.

## Introduction

Our ever-growing understanding of the human genome has revolutionized advances in cancer biology. The human transcriptome is large, including both protein-coding mRNAs and noncoding RNAs^1^. Long noncoding (lnc) RNAs, ranging from 200 nucleotides to 100 kilobases, are pervasively transcribed throughout the genome and participate in a wide array of cellular processes, particularly through *cis-* or *trans-*regulation of gene expression at enhancers, chromatin remodeling, and post-transcriptional regulation of mRNA processing^2–4^.

An expanding body of evidence points to lncRNAs as mediators of tumorigenesis in multiple types of cancer, and lncRNAs may represent a new class of targets in cancer therapy^5^ The lncRNA MALAT1 (metastasis-associated lung adenocarcinoma transcript 1) is highly expressed in non small-cell lung cancer cell lines and contributes to tumor invasion and metastasis^6^. Inhibition of MALAT1 in the MMTV–PyMT mouse model of breast cancer resulted in highly differentiated primary tumors and a nearly 80% reduction in lung metastasis^7^. The lncRNA PVT1 (plasmacytoma variant translocation 1) is expressed from the *PVT*1 gene located adjacent to MYC on human chromosome 8q24 and is coamplified with MYC in 98% of cancers^8,9^. PVT1 lncRNA regulated MYC protein and increased cell proliferation and tumorigenicity in cells with MYC amplifications^9^.

To gain a better understanding of the contribution of lncRNA to human cancer, especially different molecular subtypes of breast cancer, we examined differentially expressed lncRNA in breast cancer. Triple-negative tumors, lacking estrogen receptor (ER), progesterone receptor (PR), and epidermal growth factor receptor-2 (HER2) amplification, make up 15%-20% of all breast cancer cases^10^. TNBC more often affects younger women, women of African descent, and women with *BRCA*1 mutations^10–13^. TNBC is challenging to treat because of its heterogeneity and paucity of defined molecular targets^10^. Though patients with TNBC have a higher response rate to neoadjuvant chemotherapy than patients with receptor positive breast cancer, those who do not achieve pathologic complete response tend to relapse and develop distant metastatic disease. Additionally, triple-negative tumors often present at higher grades at diagnosis and display aggressive clinical behavior^10,11^. As a result, TNBC is associated with poor prognosis, recurrence, and shorter survival^10,11,14^.

TNBC, clinically defined by tumor receptor status based on immunohistochemistry and fluorescence *in situ* hybridization, can be further divided into molecular subgroups by gene expression signature. The majority of triple-negative tumors fall under the basal-like breast cancer (BLBC) molecular subtype; about 75% of TNBCs are classified as basal-like based on gene expression profiling, while the other 25% cluster with other mRNA subtypes (luminal A, luminal B, HER2-enriched or normal breast-like)^10–13,15,16^. Likewise, approximately 80% of BLBCs are negative for ER, PR and HER2^15^. Similar to TNBC, BLBC is a heterogeneous disease leading to a wide range of clinical outcomes; those patients who develop complete response to chemotherapy have excellent outcomes whereas the remaining patients with non-responsive tumors have the worst prognosis of all subgroups^11^.

Basal-like tumors have a high frequency of mutations in *TP53*, *RB1*, *BRCA1, PIK3CA*, and *MYC* amplification^15^. Accordingly, BLBC cells often display a highly invasive, proliferative, and dysregulated cell cycling phenotype^10^. Of note, 20% of basal-like tumors have a germline and/or somatic *BRCA1* or *BRCA2* variant, implying a significant hereditary component to BLBC^15^. Currently, there is a lack of selective agents to target basal-like tumors, leaving only chemotherapeutic options^16^. Unfortunately, this group of cancer often develops resistance to chemotherapy leading to recurrence and metastatic disease^17^. Thus, it is imperative that we better understand the unique biological drivers of BLBC in order to identify new therapeutic targets.

Here we report on the BLBC-specific lncRNA, BLAT1/RP11-19E11.1, which is involved in regulation of cell proliferation and cell death. Mechanically, this lncRNA is regulated epigenetically through CpG site methylation. We observed decreased promoter methylation and increased BLAT1 expression in a large cohort of patients with BLBC from TCGA, as well as in BLBC cell lines. There was a trend of decreased 10-year overall survival among patients with BLAT1 promoter hypomethylation. These results suggest that altered promoter methylation and expression of BLAT1 may represent a biomarker for BLBC with possible prognostic implications.

## Results

### LncRNAs are specifically expressed in basal-like breast cancer tumors

**Figure 1:**
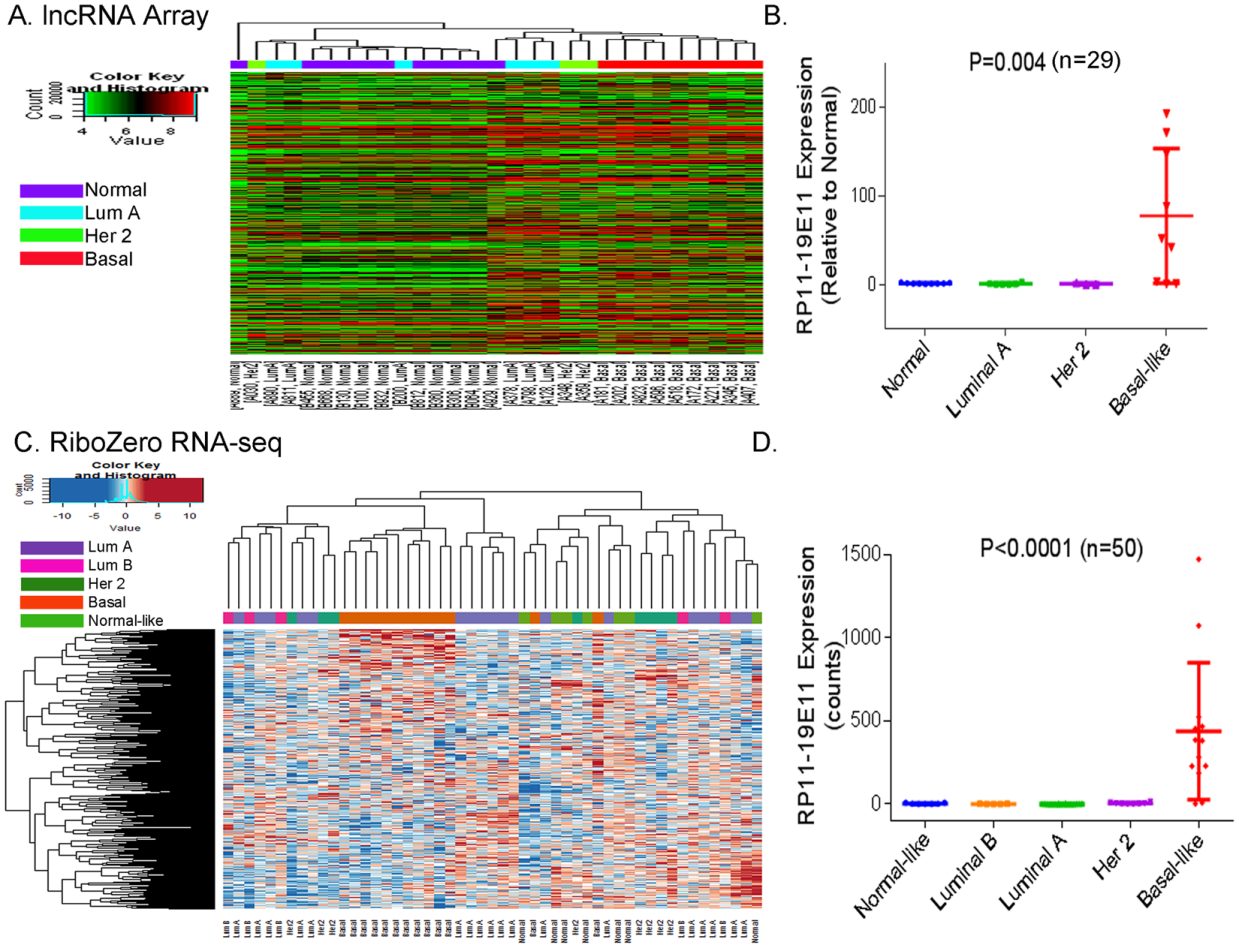
BLAT1 Expression is Highly Up-Regulated in Basal-like Breast Tumors. LncRNA expression was profiled through human lncRNA array (**A**) or RiboZero RNA-sequencing (**C**). Unsupervised hierarchical clustering of lncRNA expression shows a unique lncRNA expression signature of BLBC tumors compared to non-BLBC tumors (**A**,**C**). Both lncRNA array and RNA-seq data identified RP-11-19E11.1 (BLAT1) as a significantly up-regulated lncRNA in BLBC tumors, compared to normal breast tissues and other subtype tumors (**B**,**D**).

We applied two approaches to identify lncRNAs differentially expressed in breast cancer. First, we performed human lncRNA array using thirty breast tissues consisting of non-malignant breast tissues (n = 11) and breast tumors (n = 19). Because BLBC has a higher prevalence among African American (AA) women^18,19^, we oversampled AA women in the study (Supplementary Table 1). Unsupervised hierarchical clustering of lncRNA expression showed a different lncRNA expression signature of BLBC tumors compared to non-BLBC tumors or normal breast tissues (Fig. 1A). Among the top twenty lncRNAs specifically expressed in BLBC tumors (Supplementary Table 2), RP-11-19E11.1 represents a significantly up-regulated lncRNA in BLBC tumors, compared to normal breast tissues and tumors of other subtypes (p = 0.004) (Fig. 1B). Based on its high expression and association with BLBC tumors, we named it as Basal-Like breast cancer Associated Transcript 1 (BLAT1).

For a validation set, we conducted rRNA-depletion based RNA sequencing (Ribo-Zero RNA-seq) on fifty breast tumors from diverse patients including 66% African Americans (Supplementary Table 1). Ribo-Zero method was chosen to allow the analysis of both coding and non-coding transcripts. We confirmed a signature of differentially expressed lncRNAs in BLBC tumors compared to non-BLBC tumors (Fig. 1C). A significant up-regulation of BLAT1 was again observed in BLBC tumors compared to other tumor subtypes (p < 0.0001) (Fig. 1D). Based on the two data sets, we concluded that BLAT1 is specifically expressed in BLBC tumors.

### Analysis of BLAT1 expression in pan-cancers

**Figure 2:**
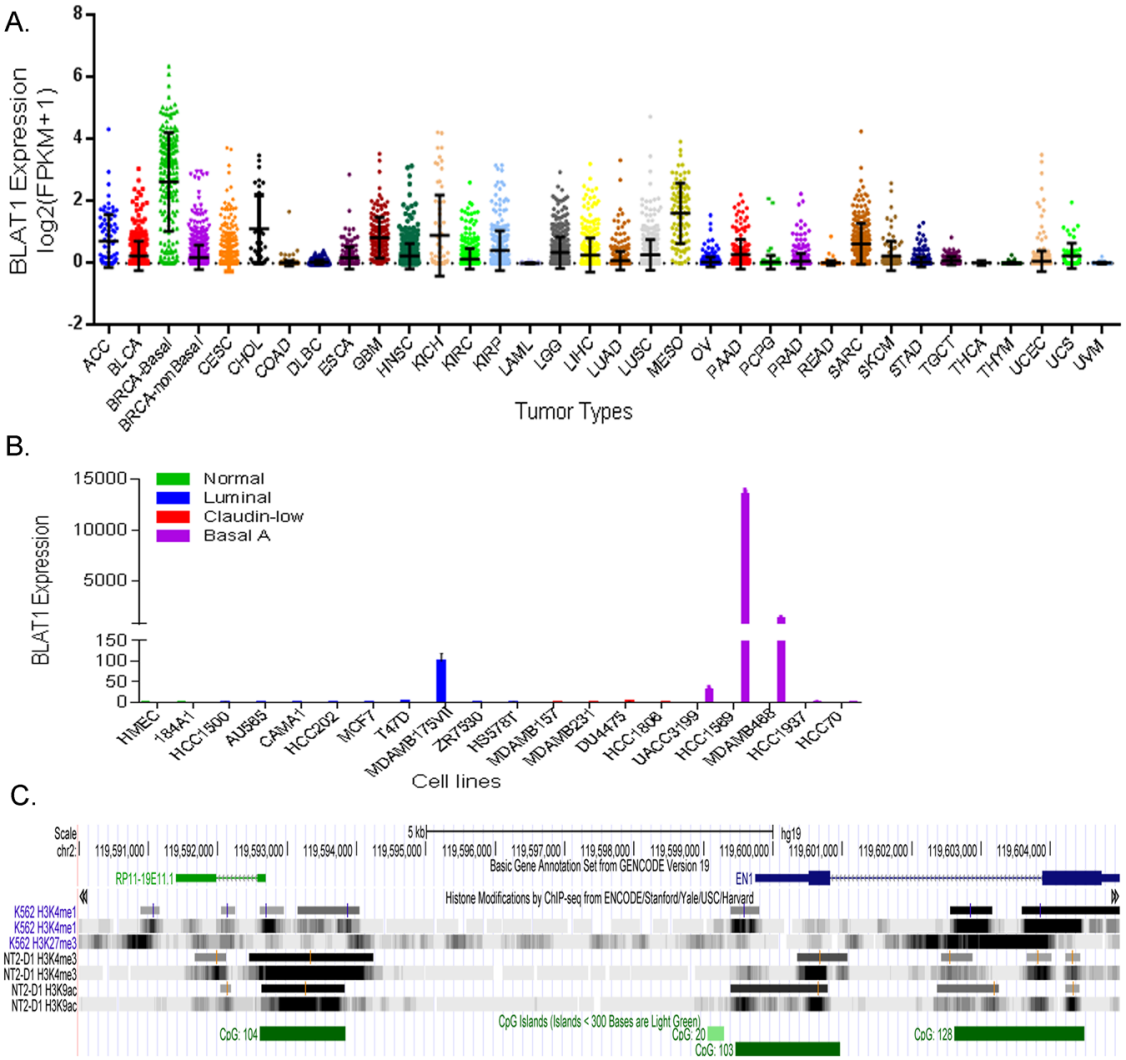
BLAT1 Expression in Pan-Cancers and Breast Cancer Cell Lines, and *In Silico* Analysis. (**A**) BLAT1 expression was analyzed using Pan-Cancer RNA-seq datasets. The values are presented as log_2_ (FPKM + 1). ACC, Adrenocortical Carcinoma; BLCA, Bladder Urothelial Carcinoma; BRCA, Breast Invasive Carcinoma; CESC, Cervical Squamous Cell Carcinoma and Endocervical Adenocarcinoma; CHOL, Cholangiocarcinoma; COAD, Colon Adenocarcinoma; DLBC, Lymphoid Neoplasm Diffuse Large B-cell Lymphoma; ESCA, Esophageal Carcinoma; GBM, Glioblastoma Multiforme; HNSC, Head and Neck Squamous Cell Carcinoma; KICH, Kidney Chromophobe; KIRC, Kidney Renal Clear Cell Carcinoma; KIRP, Kidney Renal Papillary Cell Carcinoma; LAML, Acute Myeloid Leukemia; LGG, Brain Lower Grade Glioma; LIHC, Liver Hepatocellular Carcinoma; LUAD, Lung Adenocarcinoma; LUSC, Lung Squamous Cell Carcinoma; MESO, Mesothelioma; OV, Ovarian Serous Cystadenocarcinoma; PAAD, Pancreatic Adenocarcinoma, PCPG, Pheochromocytoma and Paraganglioma; PRAD, Prostate Adenocarcinoma; READ, Rectum Adenocarcinoma; SARC, Sarcoma; SKCM, Skin Cutaneous Melanoma; STAD, Stomach Adenocarcinoma; TGCT, Testicular Germ Cell Tumors; THCA, Thyroid Carcinoma; THYM, Thymoma; UCEC, Uterine Corpus Endometrial Carcinoma; UCS, Uterine Carcinosarcoma; UVM, Uveal Melanoma. (**B**) Expression of BLAT1 was analyzed in twenty beast cell lines by qRT-PCR relative to the expression in non-malignant breast cells (184A1 and HMEC). HCC-1569 and MDA-MB-468 cells showed the highest expression among twenty cell lines. (**C**) UCSC genome browser view of 2q14.2 (hg19) shows the close location (within 7 Kb) of *BLAT1* (*RP-11-19E11*.*1*) and *EN1*. *BLAT1* has a rich epigenetic landscape (ChIP-seq data from ENCODE) and CpG islands in the regulatory region.

We next analyzed BLAT1 expression in 33 types of human cancers (n = 9,811) using previously reported lncRNA profiles in pan-cancer samples of TCGA^20^ and NCI Genomic Data Commons. BLAT1 is expressed in many types of human tumors, but with the highest expression levels in BLBC tumors (BRCA-Basal), compared not only to non-BLBC tumors (BRCA-non Basal) but also to all other tumors (Fig. 2A). The data confirmed increased expression of BLAT1 in BLBC tumors, validating BLAT1 as a novel biomarker for BLBC across all human cancers.

### BLAT1 expression is upregulated in BLBC cell lines

We examined BLAT1 expression in a total of twenty breast cancer cell lines divided by molecular subtypes (Fig. 2B). Expression of BLAT1 in breast cancer cells was analyzed by qRT-PCR relative to the expression in non-malignant breast cells (184 A1 and HMEC). Basal A cell lines (n = 5) showed higher expression of BLAT1 in general, compared to non-basal cancer cell lines (n = 13). HCC-1569 and MDA-MB-468 cells showed a 13,491 ± 496 and 1,325 ± 195 fold increase compared to 184A cells, respectively. We therefore chose these two cell lines for further functional characterization of BLAT1 *in vitro*.

### Characterization of BLAT1 identified through in silico analysis

*BLAT1* (*RP-11–19E11*.*1*) is located at 2q14.2 adjacent to *EN1* (*Engrailed 1*), a transcription factor known to be exclusively overexpressed in BLBC and to contribute to survival pathways and chemotherapy resistance (16). *BLAT1* is surrounded by a rich epigenetic landscape shown by ChIP-seq data from ENCODE, including strong H3K4me3 and H3K9ac in NT2-D1 cell lines (Fig. 2C). It also contains CpG islands with 104 CpG dinucleotides in the promoter region, suggesting that epigenetic changes specific to BLBC may play a functional role in BLAT1 expression.

### Efficient Knockdown of BLAT1 in BLBC cells

**Figure 3:**
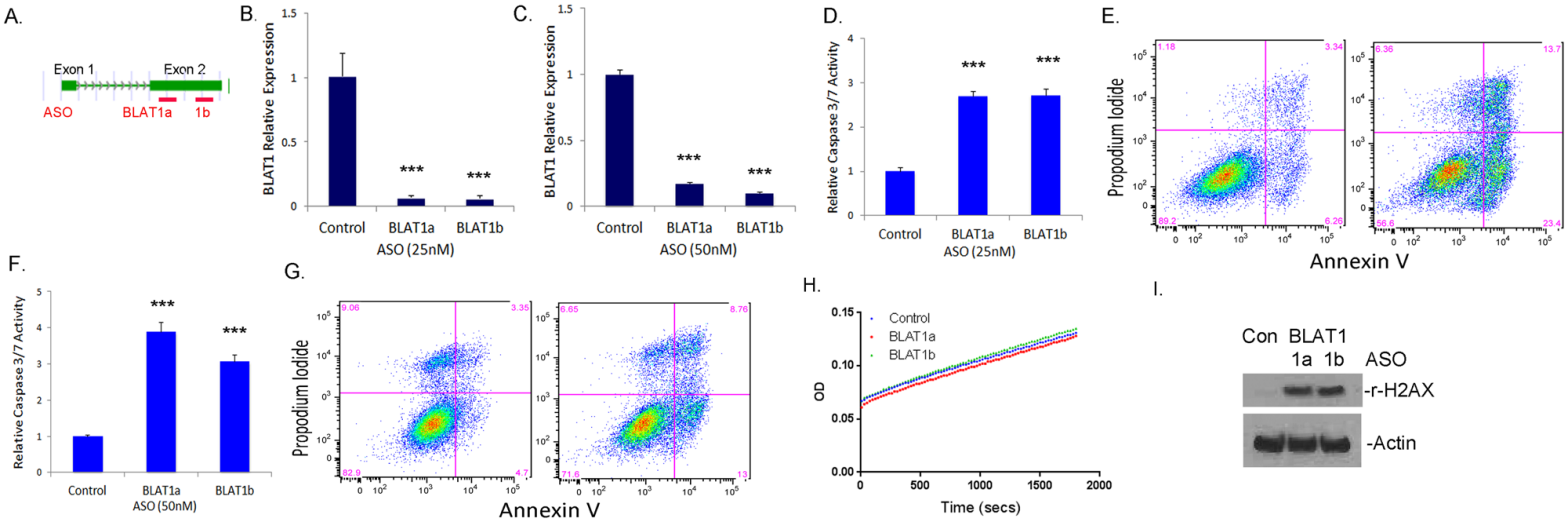
Knockdown of BLAT1 Expression Increased Apoptosis of BLBC cell lines. (**A**) Two different antisense oligonucleotides (ASO) targeting exon 2 of BLAT1 (BLAT1a and 1b, respectively) are indicated by red bars. (**B**,**C**) qRT-PCR data showed an efficient knockdown of BLAT1 expression by BLAT1a and 1b ASOs in MDA-MB-468 (**B**) and HCC-1569 cells (**C**). (**D,** **F**) BLAT1 knockdown induced significant apoptotic response in MDA-MB-468 (**D**) and HCC-1569 cells (**F**) determined by caspase 3/7 activity. (**E**,**G)** Total apoptosis was shown to be increased by flow cytometry of Annexin V/PI stained cells 3 days after ASO knockdown in MDA-MB-468 (**E**) and HCC-1569 cells (**G**). Error bars, SE (***p < 0.001 *vs*. control-ASO treated cells). (**H**) Fifty micrograms of protein were subjected to mitochondrial OXPHOS complex I activity, which is determined by the oxidation of NADH to NAD+ and the simultaneous increase in absorbance at OD 450 nm. No significant difference in the activity was observed among MDA-MB-468 cells treated with control, BLAT1a or BLAT1b ASO. (**I**) Western blot analysis showed increased expression of γ-H2AX in BLAT1a or BLAT1b ASO-treated cells, compared to the control (Con). β-Actin was used as a loading control.

We investigated the functional effect of BLAT1 knockdown on the phenotype of BLBC cells. Efficient knockdown of BLAT1 was achieved by transfecting MDA-MB-468 and HCC-1569 with locked nucleic acid (LNA)-antisense oligo nucleotides (ASOs). We designed two LNA-ASOs targeting independent regions of exon 2 of BLAT1 (Fig. 3A). Knockdown conditions were optimized separately for MDA-MB-468 and HCC-1569. Transfection of 25 nM ASO led to 94% knockdown of the BLAT1 transcript in MDA-MB-468 cells transfected with both ASO 1a and 1b compared to transfection with the ASO negative control (Fig. 3B). Likewise, 50 nM ASO resulted in 83% and 90% knockdown of the BLAT1 transcript in HCC-1569 cells transfected with ASO 1a and ASO 1b, respectively, compared to transfection with the control (Fig. 3C). None of these ASOs exhibited any inhibitory effects on neighboring gene (EN1) expression, indicating their specificities on BLAT1 lncRNAs (Supplementary Fig. 1A).

### Knockdown of BLAT1 expression increased apoptosis of BLBC cell lines

Because we observed cell death and a decrease in cell proliferation after BLAT1 knockdown, we assayed cleavage products of caspase-3 and -7 in MDA-MB-468 and HCC-1569 cells after ASO treatment. BLAT1 knockdown induced a significant increase in apoptosis in both cell lines. Caspase 3/7 activity levels were 2.70 ± 0.11 and 2.72 ± 0.013 fold higher in MDA-MB-468 cells transfected with ASO 1a and 1b, respectively, compared to cells transfected with the control ASO (Fig. 3D). Likewise, HCC-1569 cells demonstrated caspase 3/7 luminescence that was 3.89 ± 0.26 and 3.06 ± 0.18 fold higher when transfected with ASO 1a and 1b, respectively, than caspase 3/7 levels of HCC-1569 cells transfected with the control ASO (Fig. 3F). To confirm the caspase 3/7 results, we additionally quantified apoptosis with flow cytometry of annexin V-Alex488 and PI stained ASO-transfected MDA-MB-468 cells. BLAT1 knockdown resulted in a higher proportion of annexin V-positive cells compared to MDA-MB-468 cells that were transfected with the control ASO (37.1% versus 9.6%) (Fig. 3E). Flow cytometry of HCC-1569 cells also confirmed a higher proportion of annexin V-positive cells compared to HCC-1569 cells transfected with the control ASO (21.8% versus 8.1%) (Fig. 3G).

### Depletion of BLAT1 increased the DNA damage response

To determine the mechanism of how BLAT1 knockdown drove apoptosis and cell death, we first examined changes in mitochondrial enzyme activity in MDA-MB-468 cells. This is partly because the adjacent gene, *EN1*, is known to contribute to survival pathways by regulating mitochondrial activity in neurons^21^ as well as breast cancer^17^. However, when we measured mitochondrial OXPHOS Complex I enzyme activity in MDA-MB-468 cells, we did not find any difference in the activity between the control and BLAT1 ASO treated cells (Fig. 3H). We next examined aberrations in the DNA damage pathway using γ-H2AX, a marker of DNA double strand breaks^22^. Western blot analysis showed a clear increase in γ-H2AX in MDA-MB-468 cells treated with BLAT1 1a or 1b ASO, compared to the control (Fig. 3I) (Supplementary Fig. 2). The results suggest that BLAT1 knockdown induces apoptosis, partly through an increase in DNA double strand breaks.

### Hypomethylation of the BLAT1 and EN1 promoters in Basal-like tumors

**Figure 4:**
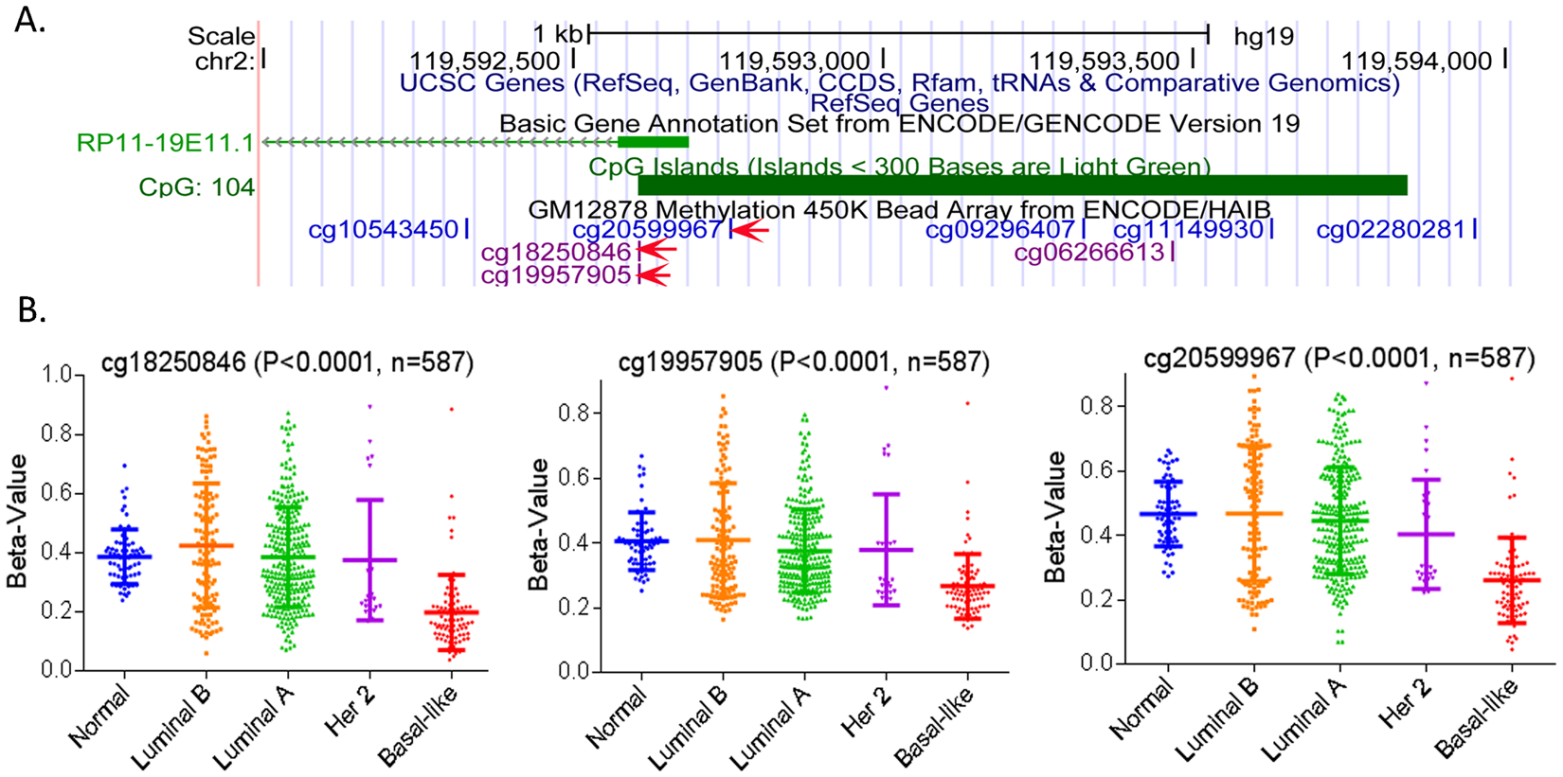
Hypomethylation of the BLAT1 Promoter in Basal-like Breast Tumors. (**A**) BLAT1 has 104 CpG sites in the promoter region. Three CpG sites (cg18250846, cg19957905, and cg20599967), which are located closest to the transcription start site, were selected for analyses. (**B**) Analysis of TCGA HumanMethylation450 Array data showed significantly lower methylation in the CpG sites in basal-like breast cancer (BLBC) tumors compared to normal breast tissues and other subtype breast tumors (p < 0.001). Beta values (0 to 1) are relative values increasing from hypomethylation to hypermethylation.

We tested if BLAT1 expression is regulated by CpG site methylation by analyzing methylation levels of three CpG dinucleotides (cg18250846, cg19957905, and cg20599967) using the TCGA HumanMethylation450 Array data (Fig. 4A). These results are expressed as beta values (0 to 1) with increasing values from hypomethylation to hypermethylation. Out of 838 patient samples with DNA methylation profile, we selected 587 samples for the analysis, as previously described (22). One-way ANOVA and Tukey’s multiple comparison tests at the three CpG islands showed significantly lower methylation in the CpG islands of the *BLAT1* promoter in BLBC tumors compared to normal breast tissues and other subtype breast tumors (Fig. 4B). These results indicate that promoter methylation of *BLAT1* may be an epigenetic modification leading to the expression differences seen between luminal and basal-like subtypes of breast cancer.

**Figure 5:**
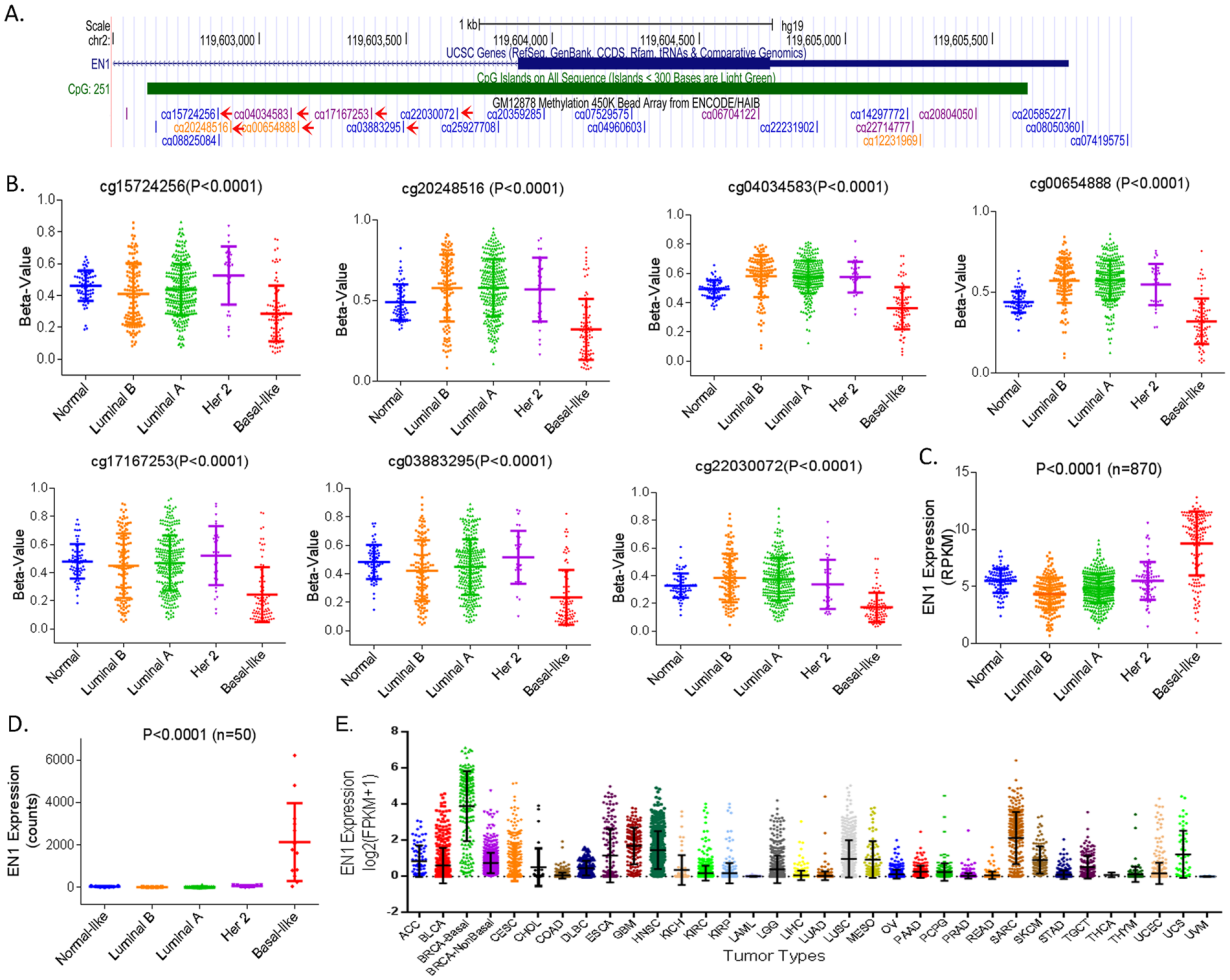
Hypomethylation of the EN1 Promoter and Increased EN1 Expression in Basal-like Breast Tumors. (**A**) Arrows indicate seven CpG sites of the EN1 promoter analyzed. (**B**) Analysis of TCGA HumanMethylation450 Array data showed significantly lower methylation in seven CpG sites of the EN1 promoter in BLBC tumors compared to normal breast tissues and other subtype breast tumors (p < 0.001). Beta values (0 to 1) are relative values increasing from hypomethylation to hypermethylation. (**C**, **D**) EN1 expression is significantly higher in basal like tumors compared to other subtype tumors, both in TCGA (**C**) and our RNA-seq datasets (**D). **(**E**) EN1 expression was analyzed using Pan-Cancer RNA-seq datasets and presented as log_2_ (FPKM + 1) values. Abbreviations of tumor types are described in Fig. 2A legend.

When we analyzed the promoter methylation of the adjacent gene, *EN1*, we also found significantly lower methylation in seven CpG sites of the *EN1* promoter in BLBC tumors, compared to normal breast tissues and other subtype breast tumors (Fig. 5 A&B). Concordantly, EN1 expression is significantly higher in basal-like tumors compared to other subtype tumors, both in TCGA (Fig. 5C) and our RNA-seq datasets (Fig. 5D). When we compared EN1 expression among pan-cancer samples, we confirmed the highest expression of EN1 in BLBC tumors compared to all other tumors (Fig. 5E). Additionally, the basal-like cell line HCC-1569 showed the highest expression of EN1 among all cancer cells (Supplementary Fig. 1B).

### Correlation between BLAT1 and EN1 methylation

Because both *BLAT1* and *EN1* are hypomethylated and highly expressed in BLBC tumors, we investigated the relationship between the two genes using TCGA methylation and RNA-seq datasets (Supplementary Fig. 1C,D). We found that methylation of the *BLAT1* CpG site, cg18250846, is significantly correlated with the methylation status at three sites in the *EN1* promoter (cg20248516, cg22030072, and cg17167253) in breast cancer (p < 0.0001) (Supplementary Fig. 1C). Similarly, expression of BLAT1 and EN1 are significantly correlated in pan-cancer samples (p < 0.001) (Supplementary Fig. 1D). The results suggest a concordant regulation of expression of both genes by DNA methylation.

### Bisulfite sequencing reveals hypomethylation of the BLAT1 promoter in BLBC cell lines

**Figure 6:**
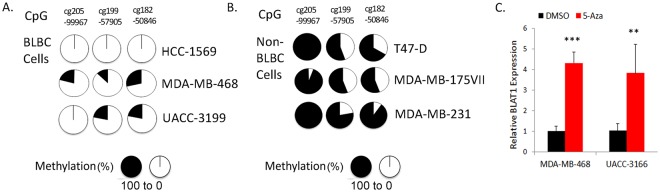
Bisulfite sequencing of the BLAT1 promoter in BLBC cell lines. (**A**,**B**) Bisulfite sequencing of three CpG sites (cg20599967, cg19957905, and cg18250846) showed a lower percentage of methylation in basal-like breast cancer (BLBC) cell lines, compared to the non-BLBC cell lines. (**C**) Inhibition of methylation with 5-Aza-2′-deoxyctidine (5′-Aza) led to a significant increase in BLAT1 expression in MDA-MB-468 and UACC-3199 cells. Error bars, SE (***p < 0.001 or **p < 0.01 *vs*. DMSO treated cells).

To further investigate the results of TCGA analysis described above, we performed bisulfite sequencing of three CpG sites (cg20599967, cg19957905, and cg18250846) within the promoter region of *BLAT1* (Fig. 6). A total of six cell lines were treated with bisulfite, including three basal-like cell lines (MDA-MB-468, HCC-1569, and UACC-3199) and three non-basal-like cell lines (T47D, MDA-MB-175VII, and MDA-MB-231). Ten or twenty clones from each cell line were sequenced and the average percentage of methylation levels was calculated. In general, the basal-like cell lines showed a lower percentage of methylation at the three sites studied compared to the non-basal-like cell lines (Fig. 6A,B). HCC-1569, a basal-like cell line with highest expression of BLAT1, showed 0% methylation at all three sites. MDA-MB-468 basal-like cells exhibited 21%, 13%, and 29% of methylation respectively at cg20599967, cg19957905, and cg18250846, whereas T47D non basal-like cells showed 100%, 56%, and 67% of methylation respectively. These data support our hypothesis that increased expression of BLAT1 in BLBC may be due to reduced methylation at the promoter.

### Treatment with 5-Aza-2′-Deoxycytidine increases BLAT1 expression in breast cancer cell lines

We then treated BLBC cells with 5-Aza-2′-deoxyctidine and further examined the regulation of BLAT1 expression by methylation. Two cell lines, MDA-MB-468 and UACC-3199, were chosen because they both had measurable baseline levels of BLAT1 expression and methylation. Inhibition of methylation resulted in an increase in BLAT1 expression in MDA-MB-468 (4.3 ± 0.54 fold) and UACC-3199 cells (3.8 ± 1.4 fold), compared to DMSO treated cells (Fig. 6C). This concomitant increase in BLAT1 expression in 5-Aza-2′-Deoxyctidine treated cells further suggests that BLAT1 expression maybe regulated through CpG dinucleotide methylation.

### CpG site methylation of BLAT1 is associated with clinical outcomes

**Figure 7:**
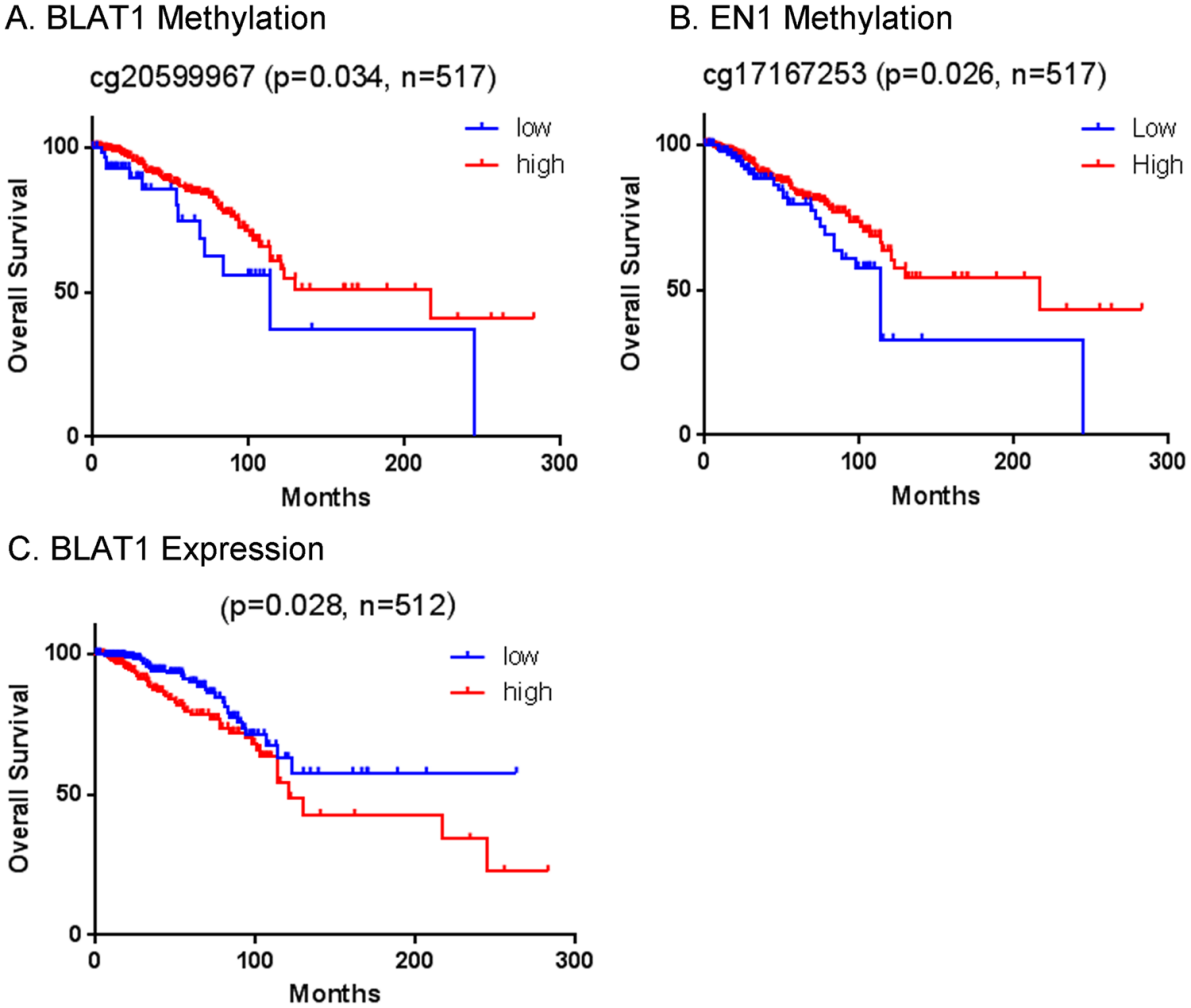
The Promoter Methylation Levels of BLAT1 and EN1 Are Associated with Clinical Outcomes. (**A**,**B**) The Kaplan–Meier estimator shows poor overall survival in patients with lower levels of the BLAT1 (beta value < 0.2) (**A**) and EN1 (**B**) promoter methylation, compared to those with higher levels of methylation (beta value > 0.2) (p = 0.034 and p = 0.026, respectively). (**C**) Patients with higher expression of BLAT1 (>median) have worse survival than those with lower expression (<median) of BLAT1 (p = 0.028).

Because BLAT1 expression plays an important role in regulating apoptosis and is epigenetically regulated, we tested an association of patient outcome with methylation levels of the *BLAT1* promoter using TCGA methylation array datasets (Fig. 7). Patients with lower levels of *BLAT1* promoter methylation, which is related to higher expression of BLAT1 in BLBC tumors, showed poor overall survival (p = 0.034) compared to those with higher methylation levels (Fig. 7A). A similar pattern was also observed in the promoter methylation of the adjacent gene, *EN1* (Fig. 7B), indicating the epigenetic significance of this locus associated with clinical outcomes.

Furthermore, when we analyzed the association of patient outcomes with BLAT1 expression levels using TCGA RNA-seq datasets, we found a significant association of higher expression of BLAT1 lncRNA with worse survival in the TCGA patient cohort (p = 0.028) (Fig. 7C). Altogether, our results indicate a potential for BLAT1 methylation and expression as a biomarker for BLBC and breast cancer prognosis.

## Discussion

BLBC, the major molecular subtype of TNBC, is a clinically challenging disease to treat due to its aggressive nature, heterogeneity, and lack of targeted therapies to date. We identified differential expression of BLAT1 in BLBC, using lncRNA array and RNA-sequencing of human breast tumors. Knockdown studies revealed that BLAT1 is functionally active in BLBC cell lines, contributing to cell survival and the DNA damage response. To gain insight into the mechanism underlying the differential expression of BLAT1 in BLBC, we compared the methylation status of the *BLAT1* promoter across the intrinsic subtypes. TCGA dataset analysis showed hypomethylation of CpG sites in the *BLAT1* promoter region that was specific to BLBC tumors. Using bisulfite sequencing, we confirmed hypomethylation of the *BLAT1* promoter in BLBC cell lines compared to non-BLBC cell lines. Inhibition of DNA methylation with 5-Aza-2′-Deoxyctidine treatment increased BLAT1 expression in breast cancer cell lines. Finally, we reported that hypomethylation of the *BLAT1* promoter was associated with worse survival in the TCGA patient cohort. These observations suggest that BLAT1 is epigenetically regulated via DNA hypomethylation and that a hypomethylation signature in BLBC leads to high levels of BLAT1 expression, contributing to aggressive clinical features.

Breast cancer subtypes appear to be associated with DNA methylation-based signatures^23,24^. A large fraction of BLBC tumors are characterized by hypomethylation events occurring within the gene body, whereas the luminal-B subtype tumors are characterized by CpG island hypermethylation events. A few selected methylation markers showed their association with clinical parameters, suggesting that these methylation markers can provide valuable information on disease prognosis in breast cancer. Our study identified a specific hypomethylation at the lncRNA promoter in BLBC tumors and its association with worse survival, strengthening the usage of methylation markers for disease prognosis, not only including protein coding regions but also including non-coding genomic regions. These markers together perhaps provide a systematic diagnostic and prognostic tool to detect and prevent breast cancer progression.

It is worthy to note that this study is based on samples mostly from AA patients. African and AA women have the highest mortality from breast cancer of all racial/ethnic groups. However, there is paucity of data on how the genomic alterations in tumors from women of diverse backgrounds interact with their physical and socio-cultural environment and how these interactions underlie the poor clinical outcomes observed in AA women. Based upon the epidemiological finding that AA women show a higher percentage of ER-negative tumors, triple-negative tumors and BLBC, we asked whether we could identify novel molecular markers for aggressive BLBC specific to AA population. We have assembled a large cohort of breast cancer cases from the diverse population of patients treated at The University of Chicago, with AA women from the South Side of Chicago making up a large proportion of these cases. We believe it is critical to continue to generate hypotheses about the biological determinants of aggressive breast cancer that disproportionately affects women of African ancestry in future studies like this.

Our results are consistent with previous findings, in which RNA-seq performed on tumors from two cohorts of breast cancer patients revealed lncRNA clustering patterns that corresponded to intrinsic subtype^25^. Bradford *et al*. found six lncRNAs to be significantly over-expressed within the BLBC subtype compared to non-BLBC, including RP11-19E11.1, whose expression correlated to EN1 upregulation in BLBC^25^. Although our study mainly focuses on lncRNAs, we also recognized the potential for the neighboring protein coding gene, *EN1*, as a biomarker for BLBC tumors. EN1 is a transcription factor shown to be overexpressed in BLBC and to contribute to survival pathways and chemotherapy resistance^14^. *EN1* is hypomethylated and upregulated in BLBC (Fig. 5), with significant correlation to *BLAT1* methylation and expression (Supplementary Fig. 1). Our data suggest a simultaneous regulation of BLAT1 and EN1 by DNA methylation in human tumors, leading to exceptionally high levels of their expression in BLBC tumors.

Interestingly, although the two genes are co-expressed in BLBC and involved in the cell survival pathway, their biological roles in the pathway seem dissimilar. EN1 regulates mitochondrial complex I activity, whereas BLAT1 knockdown increased γ-H2AX, a DNA double strand marker, with no change in mitochondrial complex I activity. Although the two adjacent genes are epigenetically co-regulated and specifically expressed in BLBC tumors, they might play distinct roles in the development of BLBC tumors, one in the DNA damage response and the other in the regulation of mitochondrial activity, which perhaps contribute to the aggressive features of BLBC tumors together.

Our report of the mechanistic regulation of a BLBC-specific lncRNA is a step forward in understanding this heterogeneous disease. While further *in vivo* studies are required before the biologic consequences of BLAT1 transcript upregulation can be extrapolated to human tumors, our *in vitro* functional analyses and the trend of decreased long-term overall survival in patients with tumors that highly expressed BLAT1 suggest that this lncRNA is biologically active and may contribute to the aggressive disease phenotype of BLBC. In the future, BLAT1 could be used as a biomarker and prognostic indicator for clinically aggressive BLBC. The functional significance of BLAT1 in BLBC cell lines makes it a potentially attractive targeted therapy. Furthermore, BLAT1 is undoubtedly just one of many lncRNAs that remain to be characterized in the complex biologic milieu of BLBC. Future study in the field of lncRNAs is necessary to expand our understanding of not only BLBC, but may also help elucidate the drivers of malignancies across the board.

## Materials and Methods

### Sample Selection

All the studies included in the lncRNA array and RNA sequencing have been approved by the Institutional Review Boards of the University of Chicago hospitals. All participants in this study provided written informed consent to allow for use of their tissue samples for research. All the methods were carried out in accordance with the guidelines and regulations of the University of Chicago. Case selection was derived from a pool of breast cancer patients who had undergone surgery at the University of Chicago hospitals. We selected female patient cases (IDC/DCIS) with frozen tissue available. Because we were interested in pre-treatment gene expression, we excluded patients who had received neoadjuvant chemotherapy.

### RNA Extraction, Sequencing, and Expression Quantification

Areas of malignant tissue were identified through light microscopy using representative top slides derived from 5μm sections of frozen tumor samples. These areas were removed using a scalpel blade and tissues were homogenized by TissueLyzer LT (Qiagen, Valencia, CA). RNA was extracted using the Qiagen AllPrep DNA/RNA/Protein mini kit protocol (Qiagen, Valencia, CA). Quality control was performed with the Agilent 4200 TapeStation system (Agilent Technologies, Santa Clara, CA). RNAs with RNA integrity number greater than 6 were selected. RNA samples were subjected to human lncRNA microarray V3 (Arraystar Inc, Rockville, MD), which includes 30,600 non-coding genes and 26,100 coding genes. For RNA-sequencing, cDNA libraries were constructed using the Illumina TruSeq Stranded Total RNA with Ribo-Zero Human kit (Illumina, San Diego, CA). RNA sequencing using 100-bp paired-end reads was performed on the Illumina HiSeq. 4000 at a depth of 80 million reads per sample. Adapter sequences were removed by Trimmomatic, alignment was performed using the Spliced Transcripts Alignment to a Reference (STAR) software and expression quantification was achieved using HTSeq. Molecular subtypes were determined by PAM50 markers, as previously described^26^.

### Cell lines and cell culture

Human mammary epithelial (HMEC) cells were purchased from Lonza (Basel, Switzerland) and breast carcinoma cell lines 184A1, AU-565, CAMA-1, DU-4475, HCC-1500, HCC-1569, HCC-1806, HCC-1937, HCC-202, HCC-70, Hs578T, MCF-7, MDA-MB-157, MDA-MB-175V11, MDA-MB-468, T-47D, UACC-3199 and ZR-75-30 were purchased from American Type Culture Collection (Manassas, VA). Cells were tested negative for mycoplasma contamination and validated for species and unique DNA profile using short tandem repeat analysis by the provider or by the authors. All cell lines were cultured in RPMI Medium 1640 (Life Technologies, Carlsbad, CA) supplemented with 10% fetal bovine serum, 1% Antibiotic-Antimycotic containing penicillin, streptomycin and Fungizone (Invitrogen, Carlsbad, CA), and 1% HEPES at 37 °C in an atmosphere containing 5% CO2.

### RNA isolation and qRT-PCR from cell lines

Total RNA was isolated from cells using RNeasy Mini Kit (Qiagen, Valencia, CA). Reverse transcription of lncRNAs and mRNAs was performed using Superscript III First Strand Synthesis kit (Invitrogen, Carlsbad, CA) with random primers. qRT-PCR was carried out in the 7900HT Fast Real-Time PCR System (Applied Biosystems, Carlsbad, CA) using TaqMan Gene Expression Assay and gene-specific TaqMan primers (Life Technologies, Carlsbad, CA). Relative quantity of expression was calculated with the ΔΔCt method using rRNA 18S as an internal control. Samples were analyzed in quadruplicate.

### Custom design of ASOs and transfection

ASOs were designed against BLAT1 using Antisense LNA GapmeRs online design tool (exiqon.com) and purchased from Exiqon (Vedbaek, DNK), along with a negative control. DNA sequences for BLAT1a and BLAT1b ASOs are 5′-AATTTGGCACGGCGG-3′ and 5′-CGGAGAAAAGGAAGTG-3′, respectively. To perform lncRNA knockdown, cells were plated in 6-well plates (5 × 10^6^ cells per well) and transfected with DharmaFECT 1 or DharmaFECT 4 (Dharmacon, Lafayette, CO) using 25 nM or 50 nM ASO per 6ul DharmaFECT reagent per well.

### Bisulfite sequencing

Bisulfite sequencing was performed on the three CpG sites (cg20599967, cg19957905, cg18250846) in the promoter region of BLAT1 in cell lines MDA-MB-468, HCC-1569, UACC-3199, T47D, MDA-MB-175VII, and MDA-MB-231. Bisulfite conversion of DNA extracted from these cell lines was performed using the EZ DNA Methylation-Gold™ Kit (Zymo Research, Irvine, CA) according to the manufacturer’s protocol. The regions with the sites of interest were amplified using ZymoTaq PCR Kit (Zymo Research, Irvine, CA) with forward primer (5′- AGGGGTTTATTTGGTGTAGGAAA- 3′) and reverse primer (5′- CTTCCCCTAATAATAAAAATAACCA- 3′) for cg20599967 and forward primer (5′- GATAGTAAGGTTGAAGAGATTTGAT- 3′) and reverse primer (5′- AAAAAACTCACCTAATACAAAAA- 3′) for cg19957905 and cg18250846. These PCR products were run on a gel to confirm specificity. The PCR products were inserted into pCR™ 4-TOPO vector using TOPO TA Cloning for Sequencing Kit (Invitrogen, Carlsbad, CA) and transformed into One Shot® TOP10 (Invitrogen, Carlsbad, CA) competent cells to grow into colonies. Plasmids were then purified using NucleoSpin® Plasmid (NoLid) Prep Kit (Machery Nagel, Germany). Sanger Sequencing was performed by the UChicago Comprehensive Cancer Center DNA Sequencing Core, using M13 primer (5′ GTAAAACGACGGCCAGT 3′). Analysis was performed by examining the chromatograms using Sequencher DNA Sequence Analysis Software (Gene Codes Corporation, Ann Arbor, MI).

### Treatment with 5-Aza-2′-Deoxyctidine

MDA-MB-468 and UACC-3199 cells were seeded at a density of 3 × 10^5^ per well of 6 well plate. One day after plating, cells were treated with 5-Aza-2′-Deoxycytidine (5-aza-CdR) at 8uM dose in DMSO and incubated for two days. Control samples were treated with the same amount of DMSO. Nucleic acids were extracted using AllPrep DNA/RNA/Protein Mini Kit (Qiagen, Valencia, CA) and subjected to bisulfite sequencing and qRT-PCR analysis using DNA and RNA accordingly.

### Apoptosis assay

Cells were plated in 6-well plates at a density of 5 × 10^5^ cells per well and transfected with ASOs for lncRNA knockdown. ASO transfected cells were cultured for 3 days, at which time apoptosis was assayed using Caspase-Glo^®^ 3/7 Reagent (Promega, Madison, WI) or flow cytometry. Caspase 3/7 activity was measured by luciferase-produced luminescent signal detected using Synergy H1 Hybrid Reader (BioTek, Winooski, VT). Flow cytometry was performed on Alexa Flour 488 annexin V and propidium iodide (PI) stained cells (Invitrogen, Carlsbad, CA) using the LSRII 3–8 (BD Biosciences, San Jose, CA).

### Mitochondrial Complex I activity assays and Western blot analysis

The mitochondrial activity assay was performed using Complex I Enzyme Activity Microplate Assay kit (ab109721) from Abcam (Cambridge, UK), according to the manufacturer’s protocol. Briefly, two days after transfection of ASOs, MDA-MB-468 cells were washed and extracted in detergent solutions. Various amounts of protein extracts (25, 50, 100, or 200 µg) were incubated with NADH and dye. The changes in absorbance were measured at OD 450 nm for 30 minutes with 20 second intervals. Western blot was performed using a standard protocol. Anti-Phospho-Histone H2A.X (Ser139) antibodies (#9718) were purchased from Cell Signaling Technology (Danvers, MA).

### The Cancer Genome Atlas (TCGA) data and statistical analysis

TCGA breast invasive carcinoma DNA methylation (Illumina Infinium HumanMethylation450 BeadChip, version 2014-05-02) datasets were extracted from UCSC Cancer Browser (https://genome-cancer.ucsc.edu/) as previously described^27^. TCGA Pan-Caner (PANCAN) RNA-seq dataset (HTSeq FPKM, version 12-11-2017) was downloaded from Genomic Data Commons (GDC) Xena Hub. We performed the one-way ANOVA and Tukey’s multiple comparison tests to compare the methylation/expression level across the breast tumor subtype groups. We included the same patient samples in the methylation (n = 516) and expression (n = 511) survival analyses, after excluding male patients, patients with no PAM50 subtype information, or patients showing discrepancy between PAM50 subtypes and pathological analyses. Statistical and survival analyses were conducted and plots were generated by GraphPad Prism 6.0 (GraphPad Software, Inc., La Jolla, CA).

## Electronic Supplementary Material

Supplementary Information

## Data Availability

The lncRNA array datasets are available through GEO (GSE119233). The RNA-seq datasets generated during and/or analyzed during the current study are available from the corresponding author on reasonable request.
