# Supplementary material for: LncRNA BLAT1 is Upregulated in Basal-like Breast Cancer through Epigenetic Modifications
Source: Sci Rep. 2018 Oct 22;8:15572. doi: 10.1038/s41598-018-33629-y (PMC6197278; doi:10.1038/s41598-018-33629-y)
Supplement: Supplementary file 1 — Supplementary Information [file 41598_2018_33629_MOESM1_ESM.docx]

Supplementary Information

**LncRNA BLAT1 is Upregulated in Basal-like Breast Cancer through Epigenetic Modifications**

Yoo Jane Han^1*^, Sonja M. Boatman^1^, Jing Zhang^1^, Xinxin C. Du^1^, Albert C. Yeh^1,2^, Yonglan Zheng^1^, Jeffrey Mueller^3^, and Olufunmilayo I. Olopade^1*^

^1^Center for Clinical Cancer Genetics and Global Health; and Section of Hematology and Oncology, Department of Medicine, University of Chicago, Chicago, IL, 60637; ^2^Fred Hutch Cancer Center, University of Washington, Seattle, WA 98109; ^3^Department of Pathology, University of Chicago, Chicago, IL, 60637

**
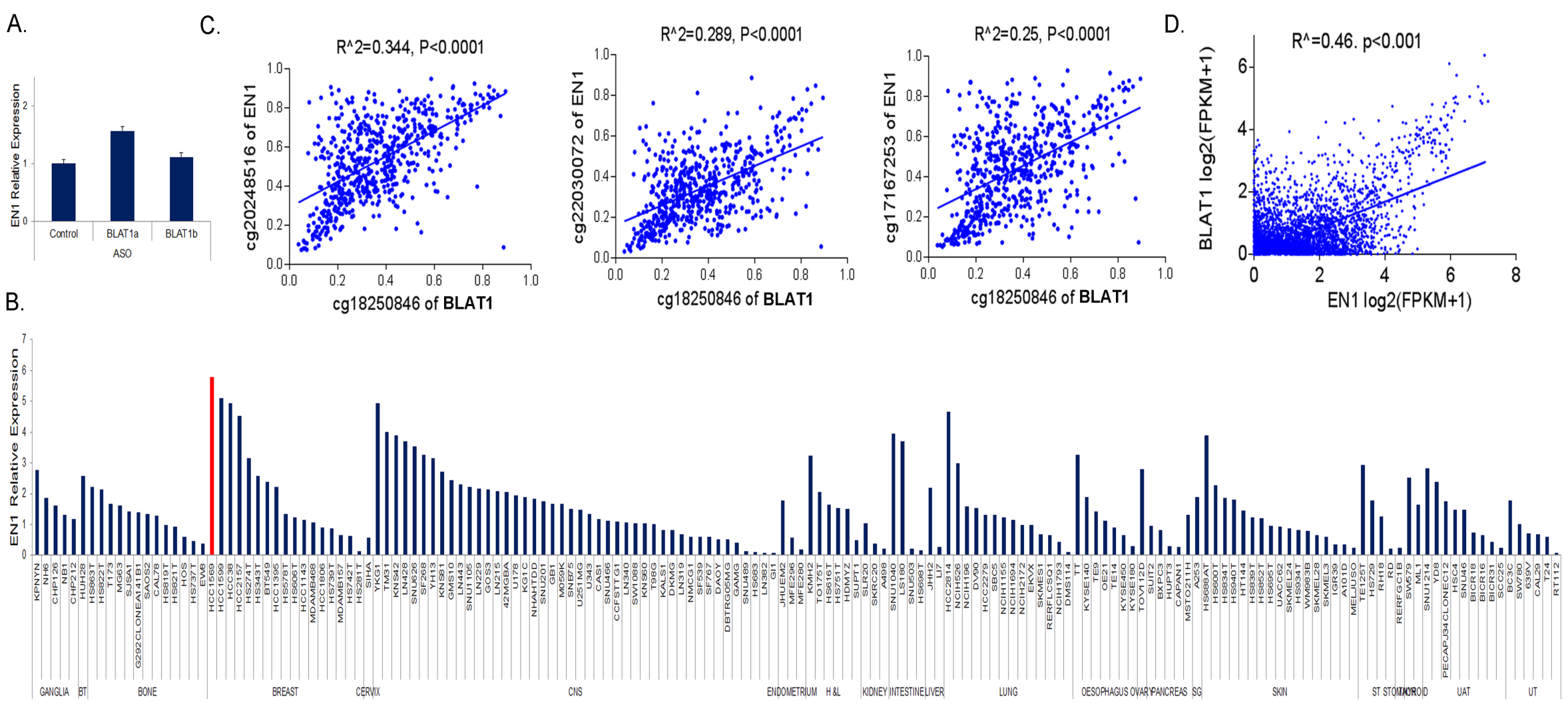
**

**Supplementary Figure 1. EN1 Expression in Cancer Cell Lines and Human Tumors.** (A) EN1 expression is not inhibited in MDA-MB-468 cells treated with BLAT1a or BLAT1b ASO, compared to control ASO treated cells. (B) CCLE dataset showed various EN1 expressions in 166 cancer cell lines, with the highest expression in a BLBC cell line (HCC-1569, indicated by the red bar). (C) The CpG site methylation at the BLAT1 promoter (cg18250846) is correlated with those at three CpG sites of the EN1 promoter. (D) BLAT1 and EN1 expressions are significantly correlated with each other across pan-cancers.

**Supplementary Figure 2.** **The Original Image of Western Blot**. The analysis showed increased expression of γ-H2AX in BLAT1a or BLAT1b ASO-treated cells, compared to the control (Con). β-Actin was used as a loading control.



**Supplementary Table 1. Clinical Features of Patients**

**
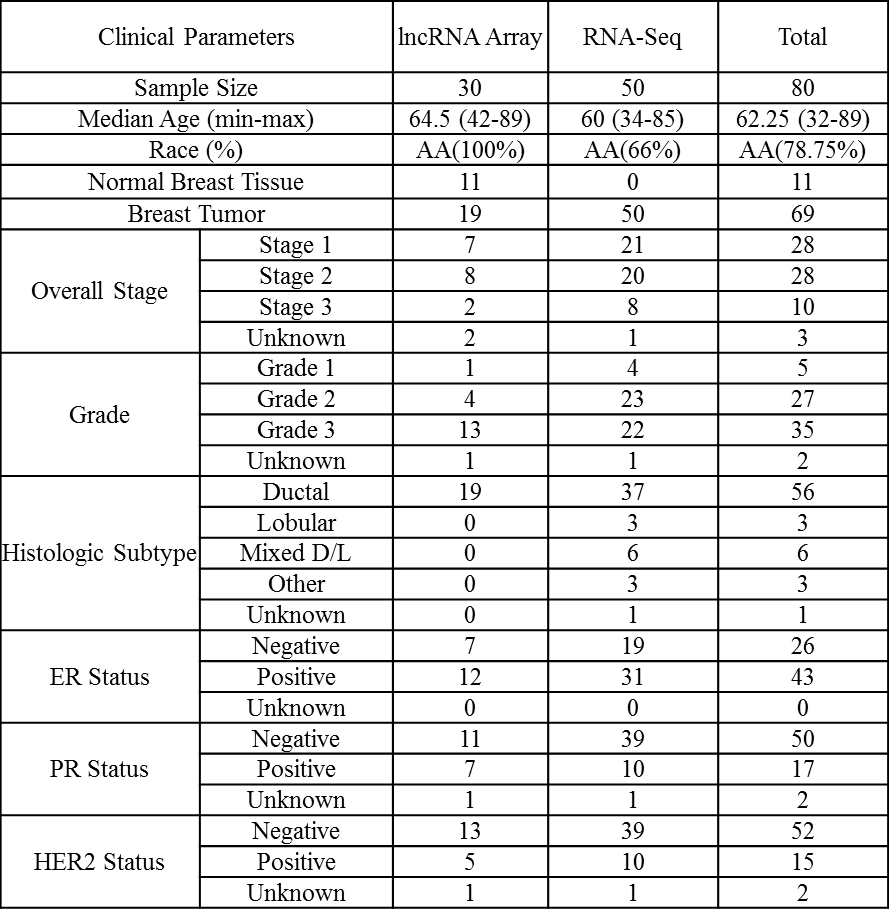
**

**Supplementary Table 2. Top Twenty lncRNAs Differentially Expressed in BLBC Tumors**

| **GeneSymbol** | **P** | **FDR** | **Fold Change** | **Regulation** |
| --- | --- | --- | --- | --- |
| BC016831 | 8.01E-08 | 6.93E-05 | 190.1094905 | down |
| RP11-20F24.4 | 1.3E-10 | 9.91E-07 | 142.57746 | down |
| BC065763 | 9E-11 | 9.91E-07 | 127.7666182 | down |
| XLOC_010998 | 4.16E-08 | 4.76E-05 | 59.9339272 | down |
| ANKRD30BP2 | 1.39E-07 | 0.000106 | 54.4082776 | down |
| CYP4Z2P | 2.51E-05 | 0.003683 | 39.4506194 | down |
| RP11-279F6.1 | 1.68E-08 | 2.56E-05 | 39.0053855 | down |
| BC070495 | 1.01E-06 | 0.000466 | 35.3111934 | down |
| AZGP1P1 | 0.000305 | 0.015296 | 31.3926704 | down |
| LOC100188947 | 2.19E-06 | 0.000766 | 26.798226 | down |
| AFAP1-AS1 | 4.25E-08 | 4.76E-05 | 122.9259873 | up |
| LA16c-83F12.6 | 0.000136 | 0.009807 | 43.6035862 | up |
| RP11-19E11.1 | 3.6E-06 | 0.001029 | 39.110817 | up |
| TCAM1P | 1.13E-06 | 0.000507 | 30.4179467 | up |
| SNHG6 | 7.5E-07 | 0.000394 | 27.5822547 | up |
| BC017578 | 7.24E-05 | 0.006647 | 20.6280096 | up |
| MGC39584 | 0.012826 | 0.091189 | 19.6739598 | up |
| AC005152.3 | 0.00969 | 0.080578 | 16.9977007 | up |
| CTD-2319I12.1 | 4.71E-05 | 0.005477 | 16.0288898 | up |
| RP11-551L14.1 | 1.86E-06 | 0.000697 | 14.7410727 | up |
